# Antibiotic Prescribing in Connection to Childbirth: An Observational Study in Two Districts in Lao PDR

**DOI:** 10.3390/antibiotics11040448

**Published:** 2022-03-25

**Authors:** Weirong Yan, Anna Machowska, Amphoy Sihavong, Vanphanom Sychareun, Kongmany Chaleunvong, Bounxou Keohavong, Jaran Eriksen, Claudia Hanson, Manivanh Vongsouvath, Annelie Brauner, Mayfong Mayxay, Sengchanh Kounnavong, Cecilia Stålsby Lundborg

**Affiliations:** 1 Department of Global Public Health, Karolinska Institutet, 171 77 Stockholm, Sweden; anna.machowska@ki.se (A.M.); jaran.eriksen@ki.se (J.E.); claudia.hanson@ki.se (C.H.); cecilia.stalsby.lundborg@ki.se (C.S.L.); 2 Vientiane Capital Health Department, Ministry of Health, Vientiane 01030, Laos; amphoys@hotmail.com; 3 Faculty of Public Health, University of Health Sciences (UHS), Vientiane 7444, Laos; vsychareun@gmail.com; 4 Institute of Research and Education Development, UHS, Ministry of Health, Vientiane 01030, Laos; kchaluenvong@gmail.com (K.C.); mayfong@tropmedres.ac (M.M.); 5 Food and Drug Department, Ministry of Health, Vientiane 01030, Laos; kbounxou@yahoo.com; 6 Department of Infectious Diseases/Venhalsan, Stockholm South General Hospital,118 83 Stockholm, Sweden; 7 Lao-Oxford-Mahosot Hospital-Welcome Trust Research Unit (LOMWRU), Microbiology Laboratory, Mahosot Hospital, Vientiane 01000, Laos; manivanh@tropmedres.ac; 8 Department of Microbiology, Tumor and Cell Biology, Karolinska Institutet, 171 77 Stockholm, Sweden; annelie.brauner@ki.se; 9 Division of Clinical Microbiology, Karolinska University Hospital, 171 76 Stockholm, Sweden; 10 Centre for Tropical Medicine and Global Health, University of Oxford, Oxford OX3 7LG, UK; 11 Lao Tropical and Public Health Institute, Ministry of Health, Vientiane 01030, Laos; sengchanhkounnavong@hotmail.com

**Keywords:** antibiotic, prescription, childbirth, Lao PDR

## Abstract

Overuse and misuse of antibiotics has frequently been reported for obstetric conditions and procedures, which may impact both the mother and the unborn baby and increase antibiotic resistance. This study aimed to investigate the antibiotic prescribing pattern in connection to childbirth in two districts in Lao PDR. It is a cross-sectional observational study. Antibiotic prescription data related to childbirth was collected via reviews of medical records in two district hospitals and five health centers in Lao PDR from September 2019 to November 2020. In total, antibiotic prescription data for 1777 women were extracted from their medical records. It was found that all women received antibiotics during in-patient care irrespective of delivery mode. When in hospital, 85.5% of the women who underwent a caesarean section got antibiotic treatment for 5 days and women who had a vaginal delivery usually had antibiotic treatment for one day or less. All the women got oral antibiotics for an additional 4–5 days upon discharge. Antibiotic prescription rate in connection to childbirth was very high in comparison with the WHO guidelines, and antibiotics were used extensively in the participating health facilities. Interventions to guide appropriate prescribing behavior in relation to childbirth are urgently needed in Lao PDR.

## 1. Introduction

Infections that occur in connection to childbirth can result in considerable morbidity and even death of the mother or her infant, especially in low- and middle-income countries (LMICs). It is estimated that obstetric infections are the reason for about 10.7% of maternal deaths worldwide [1].

There are several conditions that may increase the risk of obstetric infections among women, such as malnutrition, anemia, bacterial vaginosis, and group B streptococcal colonization [2]. Complications during delivery or childbirth or provider interventions such as operative vaginal delivery (OVD) or caesarean section may also increase the risk of infection in the postpartum period [3]. In addition, poor hygienic conditions, inadequate water and sanitation systems, lack of professional healthcare providers and high patient load can increase the risk of postpartum infections, especially in LMICs [4].

Antibiotic prophylaxis is an important intervention to reduce the risk of postpartum infections in certain cases. In Lao People’s Democratic Republic (PDR), there are several treatment guidelines regarding antibiotic prescribing/use in place at the national level, however there are no specific guidelines regarding intrapartum antibiotic use. The World Health Organization (WHO) guidelines for the prevention and treatment of maternal peripartum infections issued in 2015 recommend routine antibiotic prophylaxis for women with a third- or fourth-degree perineal tear, but not for women who underwent an episiotomy or had an uncomplicated vaginal delivery [5]. “Uncomplicated vaginal delivery” here refers to vaginal birth in the absence of any specific risk factor for or clinical signs of maternal peripartum infection [5]. In 2021, the WHO issued new guidelines recommending single-dose prophylactic antibiotics for women undergoing caesarean sections [6] as well as women undergoing OVD, which was assisted by either vacuum extractor or forceps [7].

Antibiotics are however commonly prescribed in primary care across LMICs, and a high proportion of inappropriate use has been reported [8]. Previous studies in Lao PDR also reported that unnecessary and inappropriate prescriptions of antibiotics were high [9,10,11,12]. However, the antibiotic prescribing practice during and after delivery are not well known in Lao PDR. To our knowledge, only one study that was conducted more than 10 years ago investigated the use of antibiotics in connection to childbirth in Lao PDR, and the results showed that the proportion of antibiotic use after uncomplicated vaginal delivery was high; 25% of women who delivered at home and 79% of women who delivered in hospitals received antibiotics, respectively [9].

The aim of this study was to investigate the prevalence, duration, and amount of antibiotics prescribed to women during and after delivery in two districts in Lao PDR.

## 2. Results

### 2.1. General Characteristics of the Study Subjects

In total, 1781 women delivered in the participating health facilities from September 2019 to November 2020. Among them, four medical records were excluded due to lack of complete information on delivery and antibiotic use after referral to provincial hospital. Antibiotic prescription data about the remaining 1777 women were extracted from their medical records. Of all participants, 682 (38.4%) were from Feuang district (rural district) and 1095 (61.6%) were from Vangvieng district (urban district). The mean age of the women was 24.6 ± 5.9 years of age, with 21.2% younger than 20 years of age, 63.2% between 20–30 years of age and 15.6% older than 30 years of age. Of the women, 69 (3.9%) had cesarean sections, 875 (49.2%) had uncomplicated vaginal deliveries (without any complications/episiotomy/perineal tear), 778 (43.8%) had vaginal deliveries with episiotomy or perineal tear, and the remaining 55 (3.1%) had vaginal deliveries with other conditions/complications, for example green amniotic fluid, dystocia, leaking amniotic fluid, placental abruption, premature baby, twins, or congenital malformation, based on the diagnosis information in their medical records. Of all the women, 1480 (83.3%) delivered at district hospitals and 297 (16.7%) had delivery at local health centers. In this study, we analyzed the prescription of antibiotics in three groups: (i) women with cesarean sections (CS group), (ii) women who had uncomplicated vaginal deliveries (UVD group), and (iii) women who had vaginal deliveries with episiotomy or perineal tear but without the other complications mentioned above (VD + E/P group). The remaining 55 women with other complications were not included due to the heterogeneity of complications, however, the proportion of these women was low (3.1%) (Table 1).

### 2.2. Antibiotic Prescription Rate

Regardless of the mode of delivery, all women (CS group: 69; UVD group: 875; and VD + E/P group: 778) were prescribed antibiotics during hospitalization and upon discharge.

### 2.3. Comparison of Antibiotic Prescription among Women with Different Delivery Modes

#### 2.3.1. During Hospitalization

During the hospitalization, 85.5% of the CS group women got antibiotics for 5 days, and nearly all women (99%) who had a vaginal delivery got antibiotics for one day or less. In Feuang district, women were prescribed ampicillin or amoxicillin, and in Vangvieng district, 99.8% of the UVD group got amoxicillin, and the CS and VD + E/P groups (95–98.6%) got a combination of ampicillin + gentamicin. Of the CS women, 98.6% were given intravenous antibiotics, 91.2–99.8% of the UVD women got oral antibiotics, and the VD + E/P women got antibiotics via intramuscular or intravenous or oral administration (Table 2).

#### 2.3.2. At Discharge

At discharge, all women were prescribed oral antibiotics for an additional 4–5 days in both districts. In Feuang district, 74.2–84.1% of women were prescribed ampicillin and 15.5–25.8% were given amoxicillin, and in Vangvieng district, nearly all women (99%) were given amoxicillin (Table 2).

#### 2.3.3. Antibiotic Prescription in Relation to Childbirth

The women in the CS group had the highest antibiotic prescription amount (17.54 DDDs/woman), followed by the women in VD + E/P group (8.29 DDDs/woman in Feuang district and 10.98 DDDs/woman in Vangvieng district). The women in the UVD group had the lowest amount of antibiotic prescriptions (8.18 DDDs/woman in Feuang district and 9.98 DDDs/woman in Vangvieng district) (Table 2).

### 2.4. Comparison of Antibiotic Prescriptions between District Hospitals and Health Centers

In the present study, 1480 (83.3%) women delivered at district hospitals and 297 (16.7%) had their delivery at local health centers. As cesarean section can only be conducted in Vangvieng district hospital, the comparison in this part was limited to UVD group and VD + E/P group.

#### 2.4.1. Uncomplicated Vaginal Delivery Group

During hospitalization, 99.3% of women in district hospitals and 100% of women in health centers received antibiotic treatment for one day or less. Women, however, received different antibiotics (*p* < 0.01), as 62.1% women in district hospitals received amoxicillin and 66.7% women in health centers received ampicillin. Most women in this group received oral antibiotics (district hospitals: 96.9% vs. health centers: 89.7%), however, more women in health centers received antibiotics via intramuscular route of administration (district hospitals: 2.6% vs. health centers: 10.3, *p* < 0.01). At discharge, 99.6% women in district hospitals got antibiotic prescription for 4 days, but only 53.9% women in health centers got oral antibiotics prescription for 4 days, and the rest for 5 days. There was no significant difference in the total antibiotic consumption between district hospital and health centers (*p* > 0.05) (Table 3).

#### 2.4.2. Vaginal Delivery with Episiotomy or Perineal Tear Group

During hospitalization, 99.3% of women in district hospitals and 100% women in health centers received antibiotic treatment for one day or less. Women in this group mostly received antibiotics via intramuscular route of administration (68.3% vs. 72.6%, *p* > 0.05). District hospitals and health centers used different antibiotics; 78.3% women in district hospitals received a combination of ampicillin and gentamicin, and 91.2% women in health centers used ampicillin. At discharge, most women had antibiotic prescription for 5 days, but the proportion in health centers was significantly higher than that in district hospitals (93.1% vs. 82.8%, *p* < 0.05). The amount of antibiotics prescribed in the health centers was significantly less than that in the district hospitals when calculating the total amount of antibiotics (9.31 vs. 10.35, *p* < 0.01) (Table 3).

## 3. Discussion

The present study found that all women who gave birth in the two districts received antibiotics during their hospitalization, regardless of the mode of delivery. Women who gave birth by CS typically received antibiotic treatment for 5 days, whereas those with an uncomplicated or complicated vaginal delivery got antibiotic treatment for 1 day or less. At discharge, all the women were prescribed oral antibiotics for another 4–5 days. The findings showed that the antibiotics were used to a very high extent in relation to childbirth in the studied health facilities in Lao PDR.

In Lao PDR, there were no specific guidelines regarding intrapartum antibiotic use. In the CAREChild project, our research group investigated knowledge, attitude, and practice regarding antibiotic prescription in relation to childbirth among 217 healthcare providers (HCPs) and found that only 9% of HCPs had participated in antibiotic education activities [13]. In the qualitative investigation, HCPs stated that they can obtain the related information about antibiotics from the WHO guidelines, lectures or trainings, scientific journals, or internet, however, they prescribed antibiotics following the common practice in their health facility or under the pressure of patients’ requirements instead of putting evidence-based guidelines into clinical practice [13].

It is generally agreed that routine antibiotic prophylaxis should not be given to women undergoing vaginal birth with a low-risk of infection [5,14,15]. However, the present study found that 100% of the women undergoing uncomplicated vaginal deliveries received antibiotic treatment during their hospitalization. A previous study in Lao PDR showed that 79% of the women with uncomplicated vaginal deliveries in hospitals received antibiotics [9]. In that study, the investigation was based on interviews with only 69 mothers, and no data were extracted from medical charts; therefore, the antibiotic prescription rate might have been underestimated. A study in India also revealed a high usage of antibiotics for low-risk vaginal deliveries (24–69%), and the decision of starting antibiotic was at the primary care provider’s discretion and not based on any standard departmental protocol [16], which was similar to what was found in our study. In the uncomplicated vaginal delivery group, we also found that 62.1% of women in district hospitals received amoxicillin but 66.7% of women in health centers received ampicillin, and more women in health centers received antibiotics via the intramuscular route of administration. This also reflected that HCPs prescribed antibiotics following the common practice in their health facility and based on the availability of antibiotics.

The present study also found that 100% of the women undergoing vaginal delivery with episiotomy or perineal tear received antibiotics during hospitalization. In our previous study on HCPs’ knowledge, attitude, and practice about antibiotics, about 95% agreed that they would prescribe antibiotics for vaginal delivery with episiotomy [13], which was in accordance with the findings in this study. In the latest recommendation from the WHO, it is stated that prophylactic antibiotics is needed for women undergoing OVD. On the other hand, a meta-analysis and systematic review published in 2020 concluded that the risks of postpartum infection at OVD is extremely low unless accompanied by episiotomy or third/fourth degree perineal tear [17]. A systematic review estimated that the reported incidence of childbirth-related perineal trauma wound infection ranged from 0.1–23.6% [18]. A multi-center prospective cohort study also found that episiotomy tripled the risk of infection [19]. A recently published meta-analysis which included two studies suggested that prophylactic intravenous antibiotics are effective in reducing infectious puerperal morbidities in terms of superficial and deep perineal wound infection or serious infectious complications in women undergoing OVD without clinical indications for antibiotic administration after delivery [20]. Another review showed that prophylactic antibiotics help to prevent perineal wound complications following third/fourth degree perineal tear, but it should be interpreted with caution because it only identified and included one small trial [21]. Based on the above findings, routine antibiotic prophylaxis might be necessary for women undergoing vaginal delivery with episiotomy and/or serious perineal tear. However, it is noted that there are many controversies in this field, for example, in ‘Antibiotic Treatment & Prophylaxis Guideline for Obstetrics (GL787)’ issued in 2021, routine antibiotic prophylaxis is not recommended for women with a third/fourth degree perineal tear or episiotomy [22].

In the latest WHO guideline, it is recommended that a single dose of intravenous amoxicillin and clavulanic acid should be administered as soon as possible after birth and no more than 6 h after birth for women undergoing OVD [7]. Due to the limited research evidence, the WHO has not confirmed the effects of other antibiotics. The present study found that most women who had an episiotomy or perineal tear were given oral or intramuscular antibiotics (ampicillin, amoxicillin, or a combination of ampicillin and gentamicin) and district hospitals and health centers used significantly different antibiotics. This might be due to the availability of those antibiotics in each health facility and common antibiotic prescribing practice usually followed by HCPs in their health facility. In the present study, there were no women undergoing OVD, so the antibiotic prescription pattern among OVD women in the study settings was not known.

Caesarean section is a major risk factor for maternal infection. In a Cochrane systematic review, it was shown that the use of prophylactic antibiotics in women undergoing cesarean sections reduced the incidence of wound infection and maternal serious infectious complications compared with placebo or no treatment [23]. In the present study, 3.9% of the women had a caesarean section delivery. Considering that Feuang district has no competency to do caesarean sections, the rate in Vangvieng district was 6.5%, which was higher than the cesarean section rate in Lao PDR (5.8%) reported in the Lao Social Indicator Survey II, 2017 [24]. The WHO recommends using a simple and short (single dose, 30–60 min before surgery) antibiotic regimen for prophylaxis for women with caesarean birth [6]. But the present study found that 85.5% women with caesarean section received repeated doses of antibiotics for 5 days during their hospitalization, which was much higher than the dosage and duration recommended by the WHO.

Providing repeated doses of antibiotic prophylaxis after caesarean section is a common practice in low-income countries [25,26]. However, there is growing evidence that a single dose or two doses has equal or even better effect than multiple doses of antibiotic prophylaxis (for one day or more than one day) for the prevention of post-caesarean infection also in low resource settings [25,26,27,28]. It is generally agreed that in most surgical cases, antibiotic prophylaxis should be given as a single dose and in no case should the prophylaxis time duration exceed 24 h [29]. Multiple doses of antibiotic prophylaxis will only increase the workload of HCPs, medical expense, and promote antibiotics resistance.

In addition, several studies investigated the effect of the timing of antimicrobial prophylaxis in women undergoing caesarean section. A prospective cohort study conducted in sub-Saharan Africa published in 2020 showed that the infectious problems encountered in this population would be reduced by the provision of antibiotic prophylaxis prior to the incision [30]. A Cochrane review published in 2014 including 12 trials reported that intravenous prophylactic antibiotics for cesarean section administered preoperatively significantly decreases the incidence of composite maternal postpartum infectious morbidity as compared with administration after cord clamp [31]. The WHO also recommends that it should be administrated 30–60 min before surgery [6]. All this evidence proves that there is no need to administer antibiotic prophylaxis after surgery or even to administrate it for several days. Therefore, our findings indicate that the antibiotics were also overused for women undergoing caesarean sections in the participating health facilities.

The present study also found that when discharged from health facilities, all women were prescribed oral antibiotics for another 4–5 days, mainly amoxicillin or ampicillin, regardless of the mode of delivery, which further largely increased the amount of prescribed antibiotics. Compared with district hospitals, HCPs in health centers were more likely to give 5 days of antibiotics prescription among the vaginal delivery group. It was found in our previous study that the prescription of antibiotics at discharge was given to prevent post-partum infections linked to the fear that the sterilization of the delivery room and equipment was not guaranteed, and that the health education provided would not be well understood by patients [13]. District hospitals usually have better medical conditions than health centers, which might be the reason that HCPs in health centers were more likely to give one more day of antibiotics prescription. A study from India also reported that both women who had vaginal deliveries and caesarean sections were prescribed antibiotics at discharge, but the proportion (28%) [32] was much lower compared to our study (100%).

Although evidence-based guidelines have been established to characterize specific indications, timing, duration, and type of antibiotic to be used in labor and delivery by the WHO or other organizations, the results of the present study clearly showed that these guidelines have not been adopted and implemented into clinical practice in Lao PDR. The contributory factors could include the lack of a specific national guideline regarding antibiotic prescription in relation to childbirth, HCPs having small opportunities to join antibiotic-related training [13], and HCPs usually following the common practice in their health facilities instead of guidelines [13]. It is important to take immediate actions to curb antibiotic overuse and misuse including establishing national guidelines to direct health workers, conducting educational interventions among HCPs, taking effective measures to help and promote HCPs to adhere to the guidelines, and developing a management mechanism to monitor and guide the rational use of antibiotics in health facilities.

### Strengths and Limitations

This is the first study to report on antibiotic prescription in connection to childbirth in Lao PDR based on the review of medical records. Data were collected during a period of more than one year, which we believe, provided an accurate description of the real practice of antibiotic use among HCPs in the study settings. The study included all the health facilities that provide pregnancy and delivery care service in the study areas, which can reflect the practice patterns of a large proportion of deliveries in this region. Moreover, this study has provided useful information for future interventions to promote appropriate antibiotic use.

The limitation of our study is that the data collection was based on the review of medical records, so we lacked additional information about the study subjects (e.g., whether they had other risk factors for infection such as malnutrition, anemia, etc.). Information about episiotomies or perineal tears were collected but without detailed information such as the location of the episiotomy, extension of the incision, and the extent of the perineal tear, which could affect the risk of postpartum infection.

## 4. Materials and Methods

### 4.1. Study Areas

The study is part of the CAREChild (Containment of Antibiotic Resistance—measures to improve antibiotic use in pregnancy, childbirth, and young children) project conducted in Lao PDR. Lao PDR is a lower-middle-income country with one of the fastest growing economies in Southeast Asia. Over the last decade, the country’s gross domestic product has grown at an average of 7.7% per year [33]. It was reported that the maternal mortality ratio in 2017 was 185/100,000 live births and the infant mortality rate was 36.4/1000 live births in 2019 [34]. Feuang and Vangvieng districts in Vientiane province were purposively selected for the present study based on their variations in background characteristics, representing different ethnic minorities’ population. Vientiane province is in the mid-northwest of Lao PDR. Vangvieng district (urban area) has a higher economic status than the other districts, and Feuang district is a rural area. A more detailed description of the study areas has been presented previously [35].

### 4.2. Data Collection

Data was extracted from medical records in the participating health facilities. All health facilities including two district hospitals and 5 health centers providing pregnancy, childbirth, and postnatal care services in the two districts were included. The other health facilities are not able to provide childbirth care services or no women gave birth there during the study period. Vangvieng district hospital has 30 beds, Feuang district hospital has 15 beds, and the 5 health centers have 15 beds (average 3 beds/health center), and only Vangvieng district hospital has the competency to perform cesarean sections. From September 2019 to November 2020, all medical records were reviewed and information on antibiotic use during in-patient care and at discharge from the participating health facilities were extracted. In each health facility, one nurse or midwife or assistant doctor was responsible for filling in a paper form; the age of the mother, diagnosis at discharge, the generic name of the prescribed antibiotics, dose, frequency, administration route, and duration were extracted from the medical records. The paper form was checked for completeness and then translated from Lao into English and entered into the Research Electronic Data Capture (REDCap) [36] tool by the research team in Lao PDR.

### 4.3. Data Analysis

Statistical analyses were performed using SAS software version 9.4 (SAS Institute Inc., Cary, NC, USA). Descriptive analyses included frequencies for categorical variables and mean and standard deviation for continuous variables. The Chi-square (×2) test and Fisher’s exact test were used to compare categorical variables.

The Anatomical Therapeutic Chemical (ATC) classification system and defined daily dose (DDD) was used to calculate the amount of the prescribed antibiotics [37,38]. In the ATC classification system, the active substances are divided into different groups according to the organ or system on which they act and their therapeutic, pharmacological, and chemical properties [39]. Drugs are classified into groups at five different levels [39]. The DDD is the assumed average maintenance dose per day for a drug used for its main indication in adults. Only one DDD is assigned per route of administration within an ATC code [40]. The main purpose of the ATC/DDD system is as a tool for presenting drug utilization statistics with the aim of improving drug use [37]. For this study, the total DDDs/woman was used to present the prescribing amounts of antibiotics for each woman in connection to childbirth.

## 5. Conclusions

The antibiotics in connection to childbirth were evidently overused compared to international guidelines in the participating health facilities in Lao PDR. Future studies are needed to investigate the associated factors with the prescribing behavior in relation to childbirth among HCPs in Lao PDR and develop and implement effective interventions to reduce their unnecessary antibiotic prescribing, which could help contain antibiotic resistance and reduce adverse effects of the antibiotics in pregnant women and infants.

## Figures and Tables

**Table 1:** General characteristics of the women who delivered at health facilities in Feuang and Vangvieng districts.

Variable	Group	Total *n* (%)	Feuang *n* (%)	Vangvieng *n* (%)	*p*
Number		1777 (100.0)	682 (38.4)	1095 (61.6)	
Age *^a^	<20	366 (21.2)	151 (23.5)	215 (19.9)	0.12
	20–30	1088 (63.2)	402 (62.5)	686 (63.5)	
	>30	269 (15.6)	90 (14.0)	179 (16.6)	
Delivery	CS group	69 (3.9)	- *^c^	69 (6.5)	<0.01
Mode	UVD group	875 (49.2)	453 (66.4)	422 (38.5)	
	VD + E/P group	778 (43.8)	221 (32.4)	557 (50.7)	
	VD + other complications *^b^	55 (3.1)	8 (1.2)	47 (4.3)	

Note: *^a^ 54 missing value for the variable age (Feuang district has 39 missing values and Vangvieng district has 15 missing values); *^b^ other complications include green amniotic fluid, dystocia, leaking amniotic fluid, placental abruption, premature baby, twins, congenital malformation, etc; *^c^ Feuang district has no competency to do cesarean section. Abbreviations: CS = cesarean section; UVD = uncomplicated vaginal delivery; and VD + E/P = vaginal delivery with episiotomy or perineal tear but without other complications.

**Table 2:** Antibiotics prescription during hospitalization days and at discharge.

	Groups	Feuang		Vangvieng		
Items		UVD *n* (%)	VD + E/P *n* (%)	CS *n* (%)	UVD *n* (%)	VD + E/P *n* (%)
During the hospitalization						
Days on antibiotics						
≤1 day		448 (98.9)	220 (99.5)	3 (4.4)	422 (100.0)	551 (98.9)
2–4 days		5 (1.1)	1 (0.5)	7 (10.1)	0 (0.0)	5 (0.9)
5 days		0 (0.0)	0 (0.0)	59 (85.5)	0 (0.0)	1 (0.2)
Generic name of antibiotic						
amoxicillin		66 (14.6)	9 (4.1)	0 (0.0)	421 (99.8)	10 (1.8)
ampicillin		385 (85.0)	211 (95.5)	0 (0.0)	0 (0.0)	17 (3.1)
ampicillin + gentamicin		0 (0.0)	0 (0.0)	68 (98.6)	1 (0.2)	529 (95.0)
Other *^a^		2 (0.4)	1 (0.4)	1 (1.5)	0 (0.0)	1 (0.2)
Route of administration						
Oral		413 (91.2)	128 (57.9)	0 (0.0)	421 (99.8)	10 (1.8)
Intramuscular		37 (8.2)	84 (38.0)	1 (1.5)	1 (0.2)	452 (81.1)
Intravenous		3 (0.7)	9 (4.1)	68 (98.6)	0 (0.0)	95 (17.1)
At discharge						
Days on antibiotics						
≤3 days		2 (0.4)	2 (0.9)	0 (0.0)	0 (0.0)	0 (0.0)
4 days		360 (79.5)	121 (54.8)	0 (0.0)	422 (100.0)	0 (0.0)
5 days		91 (20.1)	98 (44.3)	69 (100.0)	0 (0.0)	557 (100.0)
Name of antibiotics						
amoxicillin		70 (15.5)	57 (25.8)	68 (98.6)	418 (99.1)	553 (99.3)
ampicillin		381 (84.1)	164 (74.2)	0 (0.0)	4 (1.0)	4 (0.7)
Other *^b^		2 (0.4)	0 (0.0)	1 (1.5)	0 (0.0)	0 (0.0)
Antibiotics DDDs/woman		8.18 (1.64)	8.29 (1.38)	17.54 (1.63)	9.98 (0.25)	10.98 (0.44)

Note: *^a^ other antibiotics included ceftriaxone, a combination of ceftriaxone + gentamicin, and erythromycin; *^b^ other antibiotics included cephalexin, erythromycin, and a combination of ampicillin + metronidazole. Abbreviations: CS = cesarean section; UVD = uncomplicated vaginal delivery; and VD + E/P = vaginal delivery with episiotomy or perineal tear but without other complications.

**Table 3:** Comparison of antibiotic prescriptions in district hospitals and health centers.

	Groups	UVD Group			VD + E/P Group		
Items		District Hospitals *n* (%)	Health Centers *n* (%)	*p*	District Hospitals*n* (%)	Health Centers *n* (%)	*p*
Number of women		680	195		676	102	
During the hospitalization							
Days on antibiotics							
≤1 day		675 (99.3)	195 (100.0)		669 (99.0)	102 (100.0)	
2–4 days		5 (0.7)	0 (0.0)		6 (0.9)	0 (0.0)	
5 days		0 (0.0)	0 (0.0)		1 (0.1)	0 (0.0)	
Generic name of antibiotic				**			**
amoxicillin		422 (62.1)	65 (33.3)		10 (1.5)	9 (8.8)	
ampicillin		255 (37.5)	130 (66.7)		135 (20.0)	93 (91.2)	
ampicillin + gentamicin		1 (0.1)	0 (0.0)		529 (78.3)	0 (0.0)	
Other *^a^		2 (0.3)	0 (0.0)		2 (0.3)	0 (0.0)	
Route of administration				**			
Oral		659 (96.9)	175 (89.7)		118 (17.5)	20 (19.6)	
Intramuscular		18 (2.6)	20 (10.3)		462 (68.3)	74 (72.6)	
Intravenous		3 (0.4)	0 (0.0)		96 (14.2)	8 (7.8)	
At discharge							
Days on antibiotics				**			*
≤3 days		2 (0.3)	0 (0.0)		2 (0.3)	0 (0.0)	
4 days		677 (99.6)	105 (53.9)		114 (16.9)	7 (6.9)	
5 days		1 (0.1)	90 (46.1)		560 (82.8)	95 (93.1)	
Name of antibiotics				**			**
amoxicillin		419 (61.6)	69 (35.4)		553 (81.8)	57 (55.9)	
ampicillin		259 (38.1)	126 (64.6)		123 (18.2)	45 (44.1)	
Other *^b^		2 (0.3)	0 (0.0)		0 (0.0)	0 (0.0)	
Antibiotics DDDs/woman		9.03 (1.30)	9.10 (2.02)		10.35 (1.43)	9.31 (1.37)	**

Note: *^a^ other antibiotics included ceftriaxone and erythromycin; *^b^ other antibiotics included erythromycin and a combination of ampicillin + metronidazole. Abbreviations: UVD = uncomplicated vaginal delivery; and VD + E/P = vaginal delivery with episiotomy or perineal tear but without other complications. *: *p* < 0.05; **: *p* < 0.01.

## Data Availability

Relevant data for this study are presented in the tables. Any further data are available upon request from the corresponding author.
